# Unusual presentation of antisynthetase syndrome: a case series and review of the literature

**DOI:** 10.1186/s13256-023-04040-7

**Published:** 2023-07-30

**Authors:** Juan Estrada-Maya, María de los Ángeles Cuellar, Lina Patricia Vargas, Carmen Cecilia Gómez, Andrés Bonilla, Pedro Felipe Burgos, Sergio Alejandro Bedoya, María Valentina Oliver, Nicolás Molano, Juan Sebastián Linares

**Affiliations:** 1 Internal Medicine, Fundación Cardioinfantil-Instituto de Cardiología, Calle 163ª#13B-60, Bogotá, Colombia; 2 School of Medicine and Health Sciences, Universidad del Rosario, Bogotá, Colombia

**Keywords:** Antisynthetase syndrome, Case report, Fever of unknown origin, Unintentional weight loss, Weakness

## Abstract

**Background:**

Antisynthetase syndrome is an inflammatory myopathy that is characterized by the presence of anti-aminoacyl-tRNA synthetase antibodies. Only 30% of those who suffer from the disease can be identified. We present three Hispanic cases of antisynthetase syndrome with unusual clinical pictures were extended myositis panel results enable disease diagnosis and treatment.

**Case presentation:**

A 57-year-old Hispanic/Latino female with an erythematous scaly plaque, unresolved fever and non-immune haemolytic anaemia in whom inpatient work-up for fever of unknown origin was positive for anti-PL12 positive myositis extended panel. A 72-year-old Hispanic/Latino male with amyopathic weakness syndrome and mechanic hands in whom impatient work-up was relevant for proximal muscle uptake and anti-PM75 and AntiPL-12 myositis extended panel. And a 67-year-old Hispanic/Latino male with progressive interstitial lung disease and unresolved fever ended in myositis extended panel positive for antiPL-7. After systemic immunosuppressor treatment, patients had favourable clinical and paraclinical responses during outpatient follow-up.

**Conclusions:**

The high variability of the antisynthetase syndrome in these cases demonstrates the importance of identification through an expanded panel and highlights the probability that this is a variable disease and that we need to include emerging molecular tests to promote the timely treatment of patients.

**Supplementary Information:**

The online version contains supplementary material available at 10.1186/s13256-023-04040-7.

## Background

Antisynthetase syndrome has been classically put within the group of inflammatory myopathies, characterized by the presence of anti-aminoacyl-tRNA synthetase antibodies [1, 2]. This syndrome has been related to a classic triad consisting of pulmonary manifestations (interstitial lung disease, ILD), myositis, and arthritis, however all three are present in only 19% of cases [3]. Also, it characterized for a later phase in which other manifestations occur, such as Raynaud’s phenomenon, mechanic's hands, and fever of unknown origin [4]. Given variability of the disease, along the absence of a standard for diagnosis, the epidemiological description of the disease has become difficult. Presentation is 2–3 times more common in women than in men and begins between the fourth and fifth decades of life [3, 4].

The characteristic antibodies (anti-aminoacyl-tRNA synthetase antibodies) are only found in at most 30% of patients with inflammatory myopathy [3]. Striking new information has reveal the role of these antibodies for profiling clinical manifestation of antisynthetase syndrome [5, 6]. ILD occurs in 86% of the cases and radiological patterns are associated with anti-Jo1 antibodies (histidyl synthetase) (70–90%), anti-PL-7 (threonyl-tRNA synthetase), anti-PL-12 (alanyl-tRNA synthetase), and anti-OJ (isoleucyl-tRNA) [5–9]. Myositis is related to the presence of anti-Jo1 in 91% of cases [10]. Cutaneous involvement, typically mechanic's hands, occurs up to 30% of cases and is directly related to the presence of anti-Jo-1 (histidyl synthetase), anti-PL-12 (alanyl-tRNA synthetase), anti-PL-7 (threonyl-tRNA synthetase), and anti-EJ (glycyl-tRNA synthetase) [11].

Considering that the heterogeneity in the onset of antisynthetase syndrome constitutes a diagnostic challenge, we present three cases. Although they had different clinical courses, use of the inflammatory myopathy autoimmunity panel allows to explain clinical presentation, make diagnosis, start treatment, and define prognosis.

## Cases presentation

### Case 1

**Fig. 1:**
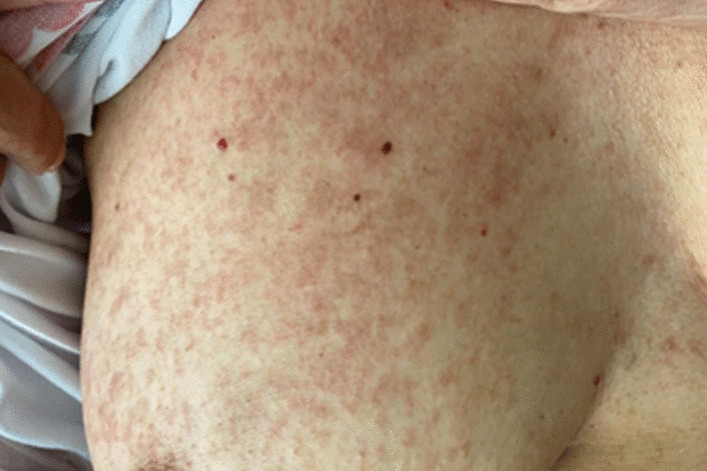
Brown, oedematous, nonpruritic, or painful plaques located on the upper trunk of patient 1

A 57-year-old Hispanic/Latino woman came to the emergency department complaining of erythematous, oedematous, scaly plaques, not pruritic or painful, initially located in the anterior region of the chest without other associated symptoms, for 1 month. Progressively, the lesions spread to the posterior thorax, arms, and legs, turning brown (Fig. 1), associated with quantified fever peaks. Previously healthy, she denied any relevant medical history, family illnesses, recent travel, or exposure to drugs or toxins. Initially, she had consulted another institution, where fever and normochromic normocytic anaemia were observed, and they referred her to us due to progression of the lesions

Upon admission, her vital signs were within normal ranges, and the physical examination was positive for the skin lesions described (Fig. 1). With the diagnostic impression of fever of unknown origin, the diagnostic algorithm suggested by Infectious Disease Society of America (IDSA) were performed, with a finding of non-autoimmune haemolytic anaemia (haemolysis profile with negative Coombs) and hyperferritinaemia (> 40,000 ng/mL) (see Additional file 1: Table S1). She had no autoimmunity stigmata, and her immune profile was negative (anti-nuclear antibodies (ANA), anti-extractable nuclear antigens antibodies (anti-ENA), anti-neutrophil cytoplasmic antibodies (ANCA), creatine phosphokinase (CPK), anti-Jo1, anti-PL, and APS). Mechanical causes of haemolysis, systemic and opportunistic infections (blood cultures, *Bartonella* serology, HIV, hepatotropes, Epstein–Barr virus, and Toxoplasma), and neoplasms were ruled out for her age group (endoscopy, colonoscopy, mammography, and cervicovaginal cytology).

A positron-emission tomography (PET) scan was performed with focal uptake in the parietal region (see Additional file 2: Fig. S1), which was rejected by brain magnetic resonance imaging (MRI) within normal ranges. The skin biopsy revealed multinucleated giant cells suggestive of cytomegalovirus, with a negative viral load, for which she received a therapeutic test with ganciclovir (5 mg/kg/d), but the symptoms persisted.

The patient still had feverish peaks and episodes of haemolytic anaemia, now associated with leukopenia and thrombocytopenia, for which a bone marrow biopsy, flow cytometry, and karyotype studies were performed, all yielding normal results. In the absence of a clear diagnosis, an expanded autoimmunity panel was requested, including autoimmune myopathies with normal CPK, electromyography, and positive anti-PL12 results (Table 1). Based on these findings, prednisolone (1 mg/kg/d) and azathioprine (50 mg/d) were started. This cured her fever, resolved her skin lesions, and normalized her cell counts. Outpatient follow-up is remarkable for resolution of the symptoms, normalization of blood counts, and corticoid weaning at 3 months Rheumatology consult, continuing with azathioprine (50 mg/d). Major events of the case are listed chronologically in Additional file 3.

**Table 1:** Myositis screening extended panel for 16 antigens

Antigen/case result	Case 1	Case 2	Case 3
Mi-2 alpha (mi-2a)	Negative	Negative	Negative
Mi-2 Beta (mi-2b)	Negative	Negative	Negative
TIF 1 Gamma (TIF1g)	Negative	Negative	Negative
MDA5 (MDA5)	Negative	Negative	Negative
NXP2 (NXP2)	Negative	Negative	Negative
SAE1 (SAE1)	Negative	Negative	Negative
Ku (Ku)	Negative	Negative	Negative
PM-Scl 100 (PM100)	Negative	Negative	Negative
PM-Scl 75 (PM75)	Negative	Positive+	Negative
Jo-1 (Jo-1)	Negative	Negative	Negative
SRP (SRP)	Negative	Negative	Negative
PL-7 (PL-7)	Negative	Negative	Positive+
PL-12 (PL-12)	Positive+	Positive+	Negative
EJ (EJ)	Negative	Negative	Negative
OJ (OJ)	Negative	Negative	Negative
Ro-52 (Ro-52)	Negative	Negative	Negative

*TIF 1 Gamma (TIF1g)* transcription intermediary factor 1-gamma, *MDA5 (MDA5)* melanoma differentiation-associated gene 5, *NXP2 (NXP2)* nuclear matrix protein 2, *SAE1 (SAE1)* small ubiquitin-like modifier (SUMO) activating enzyme 1, *PM-Scl 100 (PM100)* Polymyositis-associated gene 100, *PM-Scl 75 (PM75)* Polymyositis-associatd gene 75, *SRP (SRP)* signal recognition particle, *PL-7 (PL-7)* threonyl-tRNA synthetase, *PL-12 (PL-12)* alanyl-tRNA synthetase, *EJ (EJ)* glycine-tRNA synthetase, *OJ (OJ)* isoleucyl‐tRNA synthetase

### Case 2

**Fig. 2:**
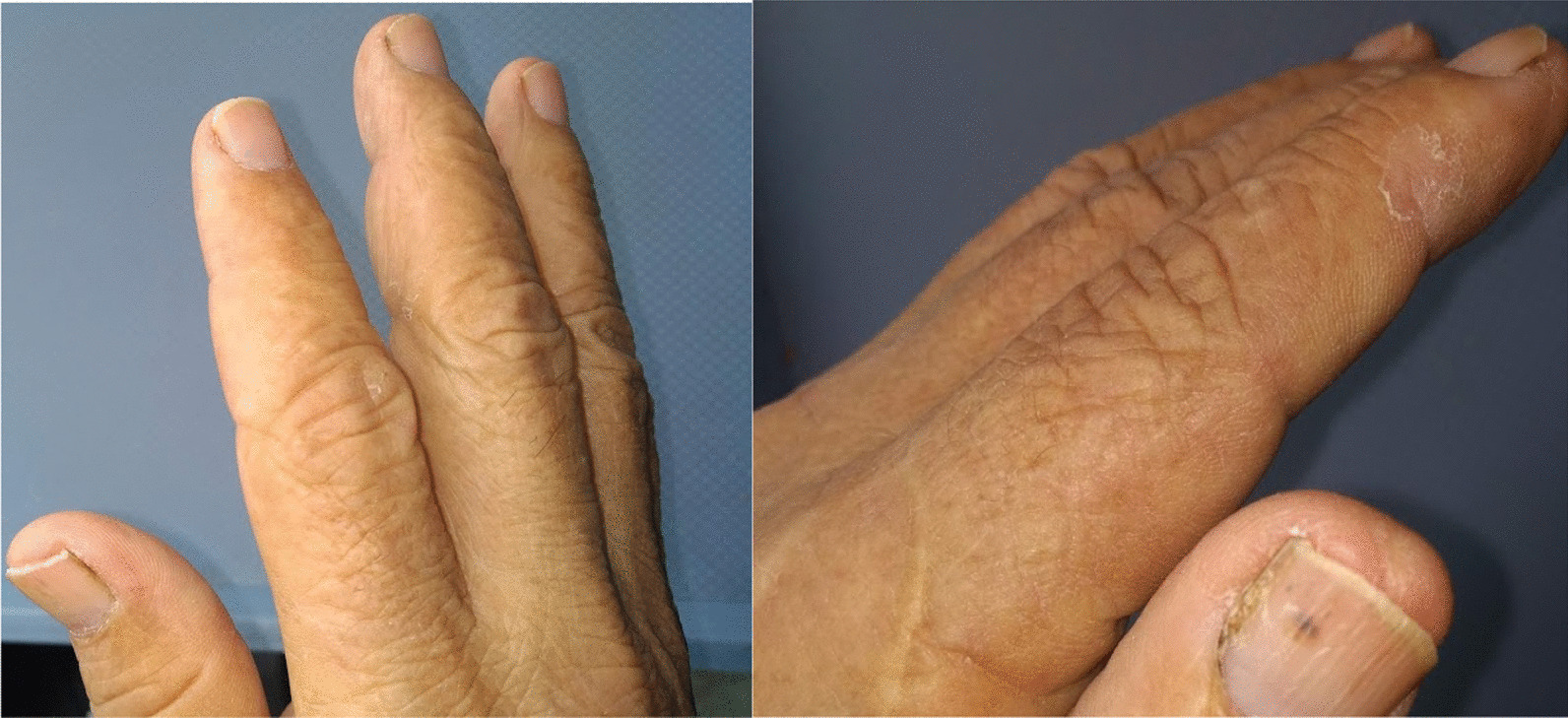
Desquamative lesion on each internal of the 3rd finger in the middle interphalangeal joint. Desquamative lesion on the inner side of the second finger at the proximal interphalangeal joint level

A 72-year-old Hispanic/Latino man, with a history of diabetes mellitus and high blood pressure, was admitted to our institution in the context of febrile of unknown origin, in addition to unintended weight loss (10 kg in 4 months) and weakness of the shoulder girdle initially in the right upper limb and then bilaterally mainly in biceps brachii and brachioradialis, joint pain in proximal interphalangeal and metacarpophalangeal joints and desquamative lesion on right hand digits (Fig. 2). Given his initial condition, we wanted to rule out inflammatory myopathy. His CPK values were normal, and electromyography and neuroconduction studies showed entrapment of the median nerve as the only finding. We decided to expand our study with an infectious profile, consisting of blood cultures, serology *Bartonella* spp., Rickettsia, Leptospira, Brucella, histoplasma, HIV, Epstein–Barr virus hepatotropes, toxoplasma, thick blood film, and dengue (given the region of origin), all of which were negative; an autoimmunity profile (ANA: positive 1:160, homogeneous pattern; negative for anti-ENA, rheumatoid factor (RF), SCL 70, and JO-1), which was a negative result; and as a study of neoplastic aetiology, upper digestive endoscopy (without alterations), colonoscopy (Kudo II polyp negative for malignancy), and computerized tomography (CT) of the chest and abdomen with contrast (suggesting lymphadenopathy in the mediastinum) (see Additional file 1: Table S1).

**Fig. 3:**
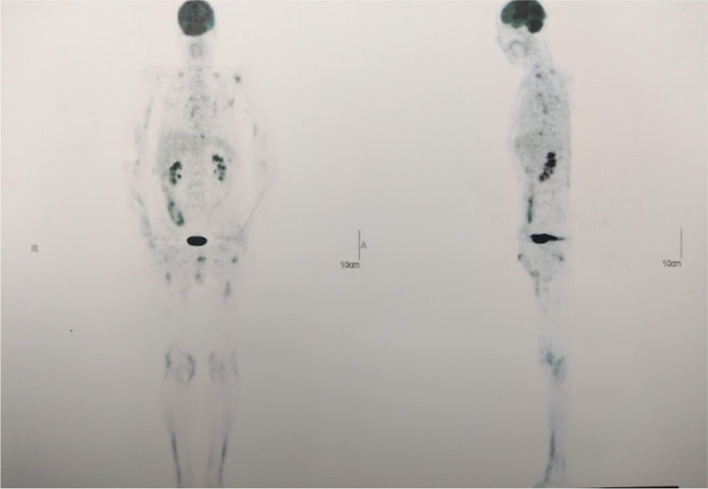
The diffuse and patchy increase in the metabolic activity of some muscles in the upper limbs and both thighs as well as the gluteal region is striking. Anteroposterior and lateral views

Due to the persistence of fever, IDSA diagnostic algorithm for fever of unknown origin was used to perform PET-CT, which did not show hyperuptake in previously described lymphadenopathies but showed hypermetabolic uptake in the upper limbs and buttocks (Fig. 3). Expanded panel of inflammatory myopathy with positivity for anti-PM75 and anti-PL12 (Table 1), compatible with antisynthetase syndrome. Management was started with cyclosporine (50 mg every 12 h) and steroids (initially methylprednisolone 500 mg for 3 days and then prednisolone 20 mg/d), with improvement in muscle weakness and febrile syndrome. Outpatient follow-up after 1 year, the patient reports improvement in weakness and being able to perform activities as before hospitalization, but there are no diagnostic tests. Major events of the case are listed chronologically in Additional file 3.

### Case 3

A 67-year-old Hispanic/Latino man with a history of organized cryptogenic pneumonia-type ILD diagnosed by transbronchial biopsy in 2014 in our institution, with negative secondary studies (autoimmune, exposure and neoplasia profile) previously treated with systemic steroids and bronchodilators. Who was being follow-up in outpatient setting due dyspnea despite bronchodilators (4 months before admission) were a chest CT was remarkable for interstitial lung disease without an specific interstitial pattern, and a normal pulmonary function tests. Now he consulted for 1 month of worsening respiratory symptoms due breathlessness (dyspnea grading scale mMRC 4, previous 1), non-productive cough and fever.

**Fig. 4:**
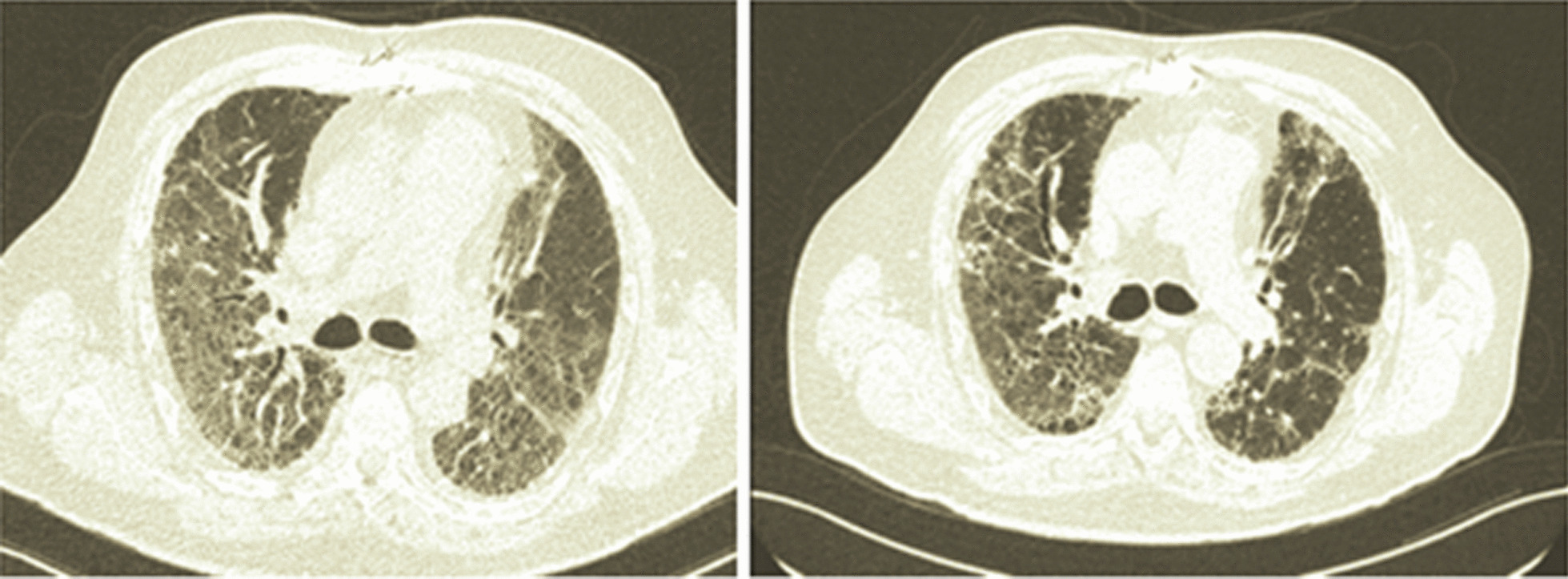
The tomographic evolution of patient 3 is shown. The image on the left shows the admission computerized tomography scan, with significant involvement of ground-glass lesions and some changes due to fibrosis. The image on the right shows computerized tomography after treatment with cyclophosphamide and steroids, with resolution of the ground-glass lesions

On admission exacerbation of drug-induced ILD vs. infectious aetiology was suspected, and new interstitial thickening and changes due to pulmonary fibrosis were documented on a high-resolution computed tomography shown in Fig. 4. We decided to start antibiotic management without improvement. Given the scenario of the COVID-19 pandemic, we took a blood sample for RT-PCR for SARS-CoV-2. He underwent fibreoptic bronchoscopy with bronchoalveolar lavage, which was negative, as well as cultures, PCR for mycobacteria tuberculosis, and the washing stains (fungi, bacteria, KOH, and cytological) (see Additional file 1: Table S1). We decided to start steroids with tomographic control at 8 days, which showed persistence of the pulmonary inflammatory process. The decision-making board decided to expand the autoimmunity study through an extended profile for inflammatory myopathies. It showed anti-PL7 positivity, compatible with antisynthetase syndrome (Table 1). Therefore, we decided to start cyclophosphamide (500 mg IV/d) and steroids (methylprednisolone 500 mg for 3 days and then prednisolone 1 mg/kg/day). Outpatient follow-up was remarkable for oxygen weaning, returns to basal dypsnea grading scale mMRC 1/4, along with the improvement of ground glass component on control CT after 12 weeks of treatment. Major events of the case are listed chronologically in Additional file 3.

## Discussion

Antisynthetase syndrome is a diagnostic challenge given its variable presentation as a spectrum of inflammatory diseases. Its name might ought to change because it probably encompasses a group of diseases. The three cases presented by our group did not have the cardinal symptoms and signs of the disease and could not even have been classified as antisynthetase syndrome without the findings of the expanded panel for myopathy. The exclusive use of anti-Jo1, the antibody most described in the literature (for our negative case in all three cases) and the most widely available in most of the institutions in our country, excludes a good percentage of scenarios where the antisynthetase syndrome is negative for this antibody [12, 13].

Baccaro *et al.* [14] conducted in Brazil a retrospective analysis of 55 patients diagnosed with antisynthetase syndrome between 2000 and 2019, a study that not only included patients with anti-Jo1 but also OJ, EJ, PL-7, and PL-12, and who had an average age of onset of symptoms of 42.3 years and age at diagnosis of 46 years, both young compared to our cases. Additionally, they found as main manifestations the development of joint pain (43.6%), muscle involvement (38.2%), and mechanic’s hands, classically described in the literature, but none of our patients presented these [14, 15].

On the other hand, Noguchi *et al.* [16] presented a descriptive study of 51 patients in Japan, in which they showed the association between muscular manifestations and the prevalence of the different antibodies in antisynthetase syndrome. Of these results, it is worth highlighting the later age of presentation (60 years), similar to that of our patients and in contrast to that of the Baccaro study. They also described the presence of anti-Jo1 (29%), anti-OJ (27%), and anti-PL-7 (24%) antibodies as those with the greatest association with muscle involvement, with the extremities showing the highest frequency of involvement (51% of patients) [16].

We highlight the presence of anti-PL7 and anti-PL12 from the myopathy panel of our patients, which usually are described as having a prevalence of 2 to 5%. Although a relevant percentage of interstitial disease is described, in our case report it was only developed by the third patient, suggesting the possibility of epigenetic phenomena that lead to variability in the phenotypic expression of the disease and that will probably allow us to better classify and understand this set of entities in the future [16, 17].

Other antibodies found in the presented cases were anti PM-Scl (75 y 100). These antibodies are found in myopathies (3–6%) and are particularly describe in cases of autoimmunity overlap myopathies/Systemic Sclerosis [18, 19]. Anti-PM-75 is associated with less pulmonary involvement and mortality, when compared against other antisynthetase antibodies like Anti-Jo-1 [20]. Also, has been described in cases of proximal weakness specially in the arms and hips [18]. Case number 2 was anti-PM-75 positive, which might stand for absence of pulmonary involvement, and hyper uptake of nuclear imagining shown in upper extremities.

Regarding interstitial disease, Henrique *et al.* [21], in a study carried out in Brazil, found a strong association between the presence of anti-PL7 (90%) or anti-PL12 (100%) and the development of interstitial disease. Although our patient 3 had a disease onset with the development of nonspecific interstitial pneumonitis, patient 1 had no compromising clinical or imaging findings, leading us to think that the disease may be different in the Colombian population than in others. Latin American citizens or, failing that, the myopathy panel could be a factor for an early diagnosis of the disease [21, 22].

## Conclusions

Certainly, antisynthetase syndrome turns out to be an entity that is still difficult to diagnose [1, 3, 4, 12]. Scientific societies and experts should boost their efforts to group or subclassify this entity into different profiles to obtain sufficiently sensitive and specific clinical and laboratory criteria, but with the necessary specificity to rule out other entities with similar nuances. In this sense, the extended panel of myopathies in the right clinical setting, can represent a resource to define the diagnosis and thus establish early management of the disease.

The clinical profile in which extended myopathy panels should be included as a diagnostic tool remain to be defined through more rigorous studies, since at present it is prescribed more by the experience of the clinician. As stated before, might be helpful in cases of unexplained fever or weakness, that might be associated with other systemic involvement, but without an identified aetiology after a rational diagnostic approach.

Finally, we consider our case report as teaching about and contributing to the medical knowledge of the broad clinical scenario of the presentation of this pathology, which forces us to reconsider current diagnostic tools and the need to include new ones. Enabling immunological phenotyping of predictive data regarding illness progression, such receptor NKp30 pathway, which has demonstrated influence on the outcomes of antisynthetase syndrome [23]. Additionally, genetic profiles aim to predict disease symptoms as well as prospective treatment targets for upcoming studies, such as the MUC5B gene, which contributes to the onset of interstitial illness [24].

## Supplementary Information

**Additional file 1: Table S1.** Comparative table of admission paraclinical variables and diagnostic aids in cases of antisynthetase syndrome. Resume all diagnostic inpatient workup results including laboratories, images, and diagnostic procedures for each case.

**Additional file 2: Figure S1.** PET-CT clinical case no. 1 with hyperuptake in the parietal region. Case no. 1 PET-CT scan, remarkable for a focal hyperuptake of tracer in parietal region with followup studies that dismiss significant diseases.

**Additional file 3.** Cases timeline’s PPT. Timeline picturing clinical course, inpatient workup, therapeutics, and outpatient follow-up of each case of antisynthetase syndrome include in the series.

## Data Availability

All data generated and analyzed during the current study are included in this published article and its additional file.

## Abbreviations

ILD: Interstitial lung disease
anti-Jo1: Anti-histidyl synthetase antibodies
anti-PL-7: Anti-threonyl-tRNA antibodies
anti-PL-12: Anti-alanyl-tRNA synthetas antibodies
anti-OJ: Anti-isoleucyl-tRNA antibodies
anti-EJ: Anti-glycyl-tRNA synthetase
IDSA: Infectious Disease Society of America
ANA: Anti-nuclear antibodies
anti-ENA: Anti-extractable nuclear antigens antibodies
ANCA: Anti-neutrophil cytoplasmic antibodies
CPK: Creatine phosphokinase
PET: Positron-emission tomography
MRI: Magnetic resonance imaging
RF: Rheumatoid factor
CT: Computerized tomography
