# Supplementary material for: Unusual presentation of antisynthetase syndrome: a case series and review of the literature
Source: J Med Case Rep. 2023 Jul 30;17:325. doi: 10.1186/s13256-023-04040-7 (PMC10387198; doi:10.1186/s13256-023-04040-7)
Supplement: Supplementary file 1 — Additional file 1: Table S1. Comparative table of admission paraclinical variables and diagnostic aids in cases of antisynthetase syndrome. Resume all diagnostic inpatient workup results including laboratories, images, and diagnostic procedures for each case. [file 13256_2023_4040_MOESM1_ESM.docx]

Additional file 1. Unusual presentation of antisynthetase syndrome, report of three cases in a hospital in Bogotá, Colombia and review of the literature

| **Table S1.** Comparative table of admission paraclinical variables and diagnostic aids in cases of antisynthetase syndrome | | | |
| --- | --- | --- | --- |
| **Study** | **Case 1** | **Case 2** | **Case 3** |
| **Complete blood count**   **Leu (10^3^ cells/µL)**  **RBC (10^6^ cells/µL)**  **Hb (g/Dl)**  **Hto (%)**  **VCM (Fl)**  **HCM (pg)**  **RDW (%)**  **PLT (10^3^ cells/µL)**  **Neu (10^3^ cells/µL)**  **Lin (10^3^ cells/µL)**  **Mon (10^3^ cells/µL)**  **Eos (10^3^ cells/µL)** | 11.30   2.79   8.25  23.0  82.5  29.6  13.7  363.000  10.000  940  330  10 | 5.32  4.52  12.6  35.5  78.6  28  12.8  299  3.04  1.21  0.2  0.85 | 13.20      45.5  15.5  89.1  29.7    166  11.30  0.75 |
| **Anti-HIV-1 and HIV-2** | 0.12 (neg) | 0.06 (neg) | NA |
| **Infectious**  **Anti-VHC**  **Ag HBs**  **Anti-VHA**  **Toxoplasma serology**  **Bartonella**  **Histoplasma** | 0.21 (neg)  0.17 (neg)  10.7 (exp)  10.2 (exp)  <1/100 (neg)  - | 0.13 (neg)  0.15 (neg)  -  -  -  Negative | NA |
| **Microbiological**  **Blood cultures**  **Mushroom cultivation**  **PCR Tuberculosis**  **LBA** | Neg  -  -  - | Neg  Neg  -  - | Neg  -  -  - |
| **Autoimmunity**  **AAN**  **ENA: Ro. La, SM, Rnp**  **FR**  **ANCA**  **Anti-Jo1**  **SAF Profile**   **Slc 70:** | Neg  5.75 (neg)  -  <15.0 (neg)  Neg  -  Dvvr Ratio 1.41 (weak positive)  - | Positives 1/80 pattern homogeneous 1/160  10.33 (neg)  -  <15.0 (neg)  -  Neg  -  - | Neg  Neg  -  <15 (neg)  Neg  Neg  -   Neg |
| **Inflammatory**  **C-reactive protein**  **VSG**  **Ferritin** | 33.47  55.0  >40.000 | 2.85  16  1363 | 5.75  - |
| **Haemolysis**  **LDH (IU/L)**  **Bilirubins (mg/Dl)**  **Haptoglobin (mg/Dl)**  **Direct coombs** | 1881  2.3  9  - | 321  0.4  -  - | 227  1  -  - |
| **Biopsies**  **1. Bone marrow**  **2. Skin**  **3. Lung** | 1. Haematopoiesis three lines, iron deposits +++/+++. 2. Interface dermatosis, nonspecific vacuolar | 1. Discreetly hypocellular, myeloerythroid ratio 3 to 1, granulocytic line and megakaryocytes present, erythroid decreased, few lymphocytes and plasma cells, iron deposits present.  2. Vasculitis in residual-phase with scratching changes. | 3. Fibrosing interstitial lung disease with a usual interstitial pneumonia pattern associated with acute lung injury, compatible with  exacerbation of NIU. |
| **Pictures**  **1. CHEST TAC**  **2. TAC ABDOMEN** | 1. Normal.  2. Normal. | 1. Scarce mediastinal lymphadenopathy.  2. Thickening of the colon. | 1. Multiple, bilateral ground glass opacities with interlobular interstitial thickening and fissures. Indirect signs of pulmonary hypertension. Mediastinal lymphadenopathy. |
| **PET-CT** | Abnormal uptake in the parietal region. | Diffuse, patchy increase in metabolic activity in MMSS and MMI. | NA |
| **Procedures**  **1. Endoscopy**  **2. Colonoscopy** | 1. Chronic erosive gastritis. | 1. Nonspecific gastritis  2. Kudo II polyp. | NA |
| **Myopathies profile** | PL-12 intensity 13 (positive +). | PM- Scl 75 (PM75) positive.  PL-12 positive. | PL-7 positive. |
