# Supplementary figures and images for: Unusual presentation of antisynthetase syndrome: a case series and review of the literature
Source: J Med Case Rep. 2023 Jul 30;17:325. doi: 10.1186/s13256-023-04040-7 (PMC10387198; doi:10.1186/s13256-023-04040-7)

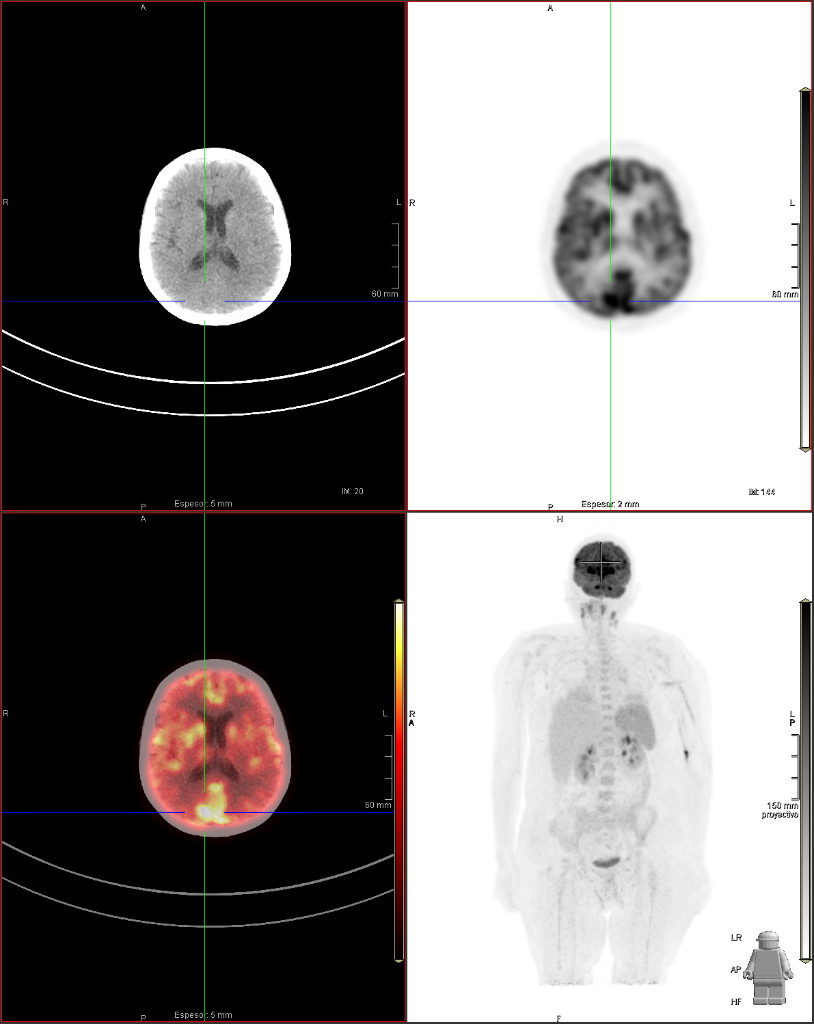

Supplement: Supplementary file 2 — Additional file 2: Figure S1. PET-CT clinical case no. 1 with hyperuptake in the parietal region. Case no. 1 PET-CT scan, remarkable for a focal hyperuptake of tracer in parietal region with followup studies that dismiss significant diseases. [file 13256_2023_4040_MOESM2_ESM.jpg]
