# Supplementary material for: Unusual presentation of antisynthetase syndrome: a case series and review of the literature
Source: J Med Case Rep. 2023 Jul 30;17:325. doi: 10.1186/s13256-023-04040-7 (PMC10387198; doi:10.1186/s13256-023-04040-7)
Supplement: Supplementary file 3 — Additional file 3. Cases timeline’s PPT. Timeline picturing clinical course, inpatient workup, therapeutics, and outpatient follow-up of each case of antisynthetase syndrome include in the series. [file 13256_2023_4040_MOESM3_ESM.pptx]

## Slide 1
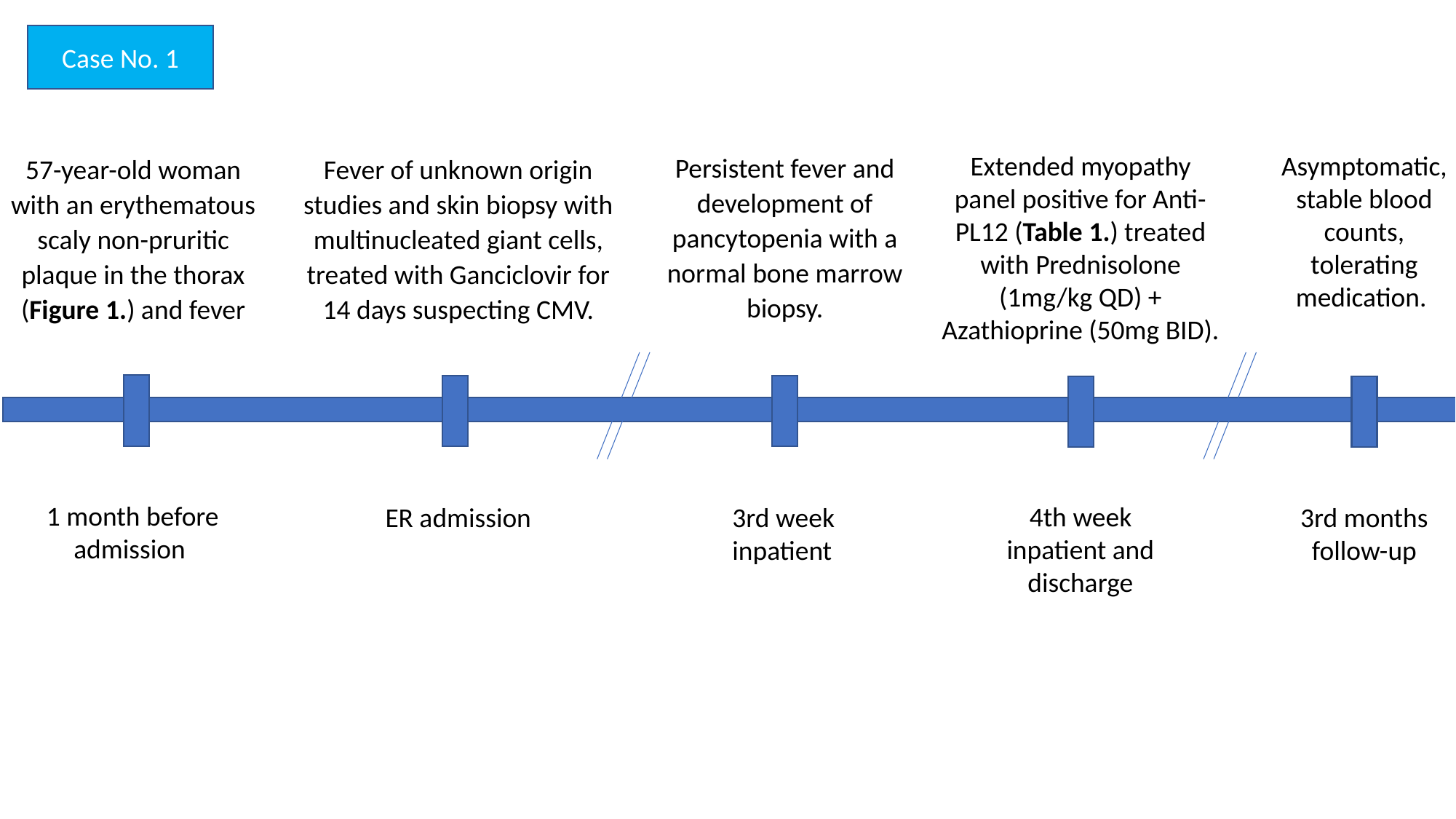

Case No. 1
Asymptomatic, stable blood counts, tolerating medication.
Persistent fever and development of pancytopenia with a normal bone marrow biopsy.
Extended myopathy panel positive for Anti-PL12 (Table 1.) treated with Prednisolone (1mg/kg QD) + Azathioprine (50mg BID).
57-year-old woman with an erythematous scaly non-pruritic plaque in the thorax (Figure 1.) and fever
Fever of unknown origin studies and skin biopsy with multinucleated giant cells, treated with Ganciclovir for 14 days suspecting CMV.
1 month before admission
4th week inpatient and discharge
ER admission
3rd week inpatient
3rd months follow-up

## Slide 2
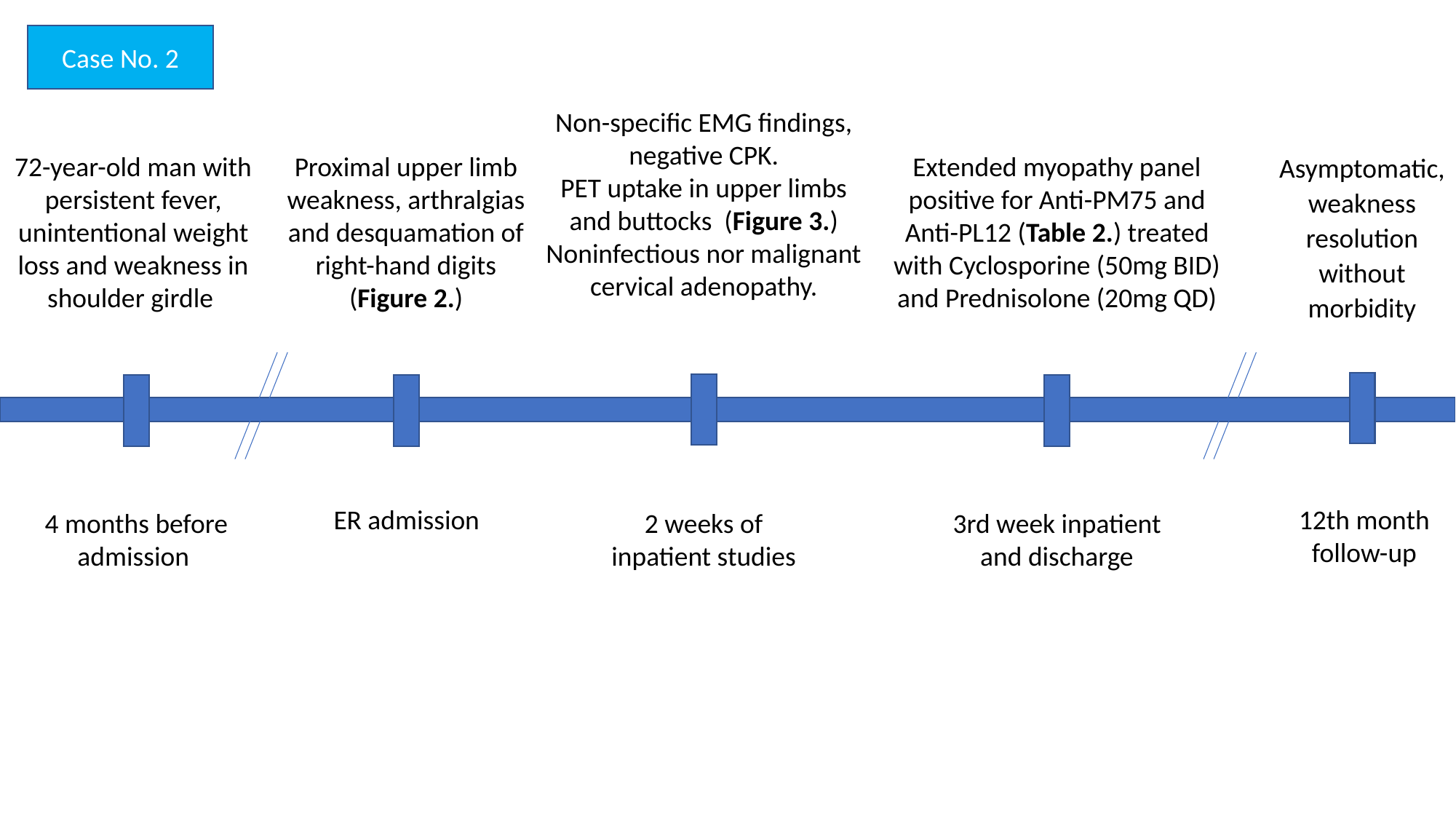

Case No. 2
Non-specific EMG findings, negative CPK.
PET uptake in upper limbs and buttocks (Figure 3.)
Noninfectious nor malignant cervical adenopathy.
Asymptomatic, weakness resolution without morbidity
72-year-old man with persistent fever, unintentional weight loss and weakness in shoulder girdle
Proximal upper limb weakness, arthralgias and desquamation of right-hand digits (Figure 2.)
Extended myopathy panel positive for Anti-PM75 and Anti-PL12 (Table 2.) treated with Cyclosporine (50mg BID) and Prednisolone (20mg QD)
ER admission
12th month follow-up
2 weeks of inpatient studies
3rd week inpatient and discharge
4 months before admission

## Slide 3
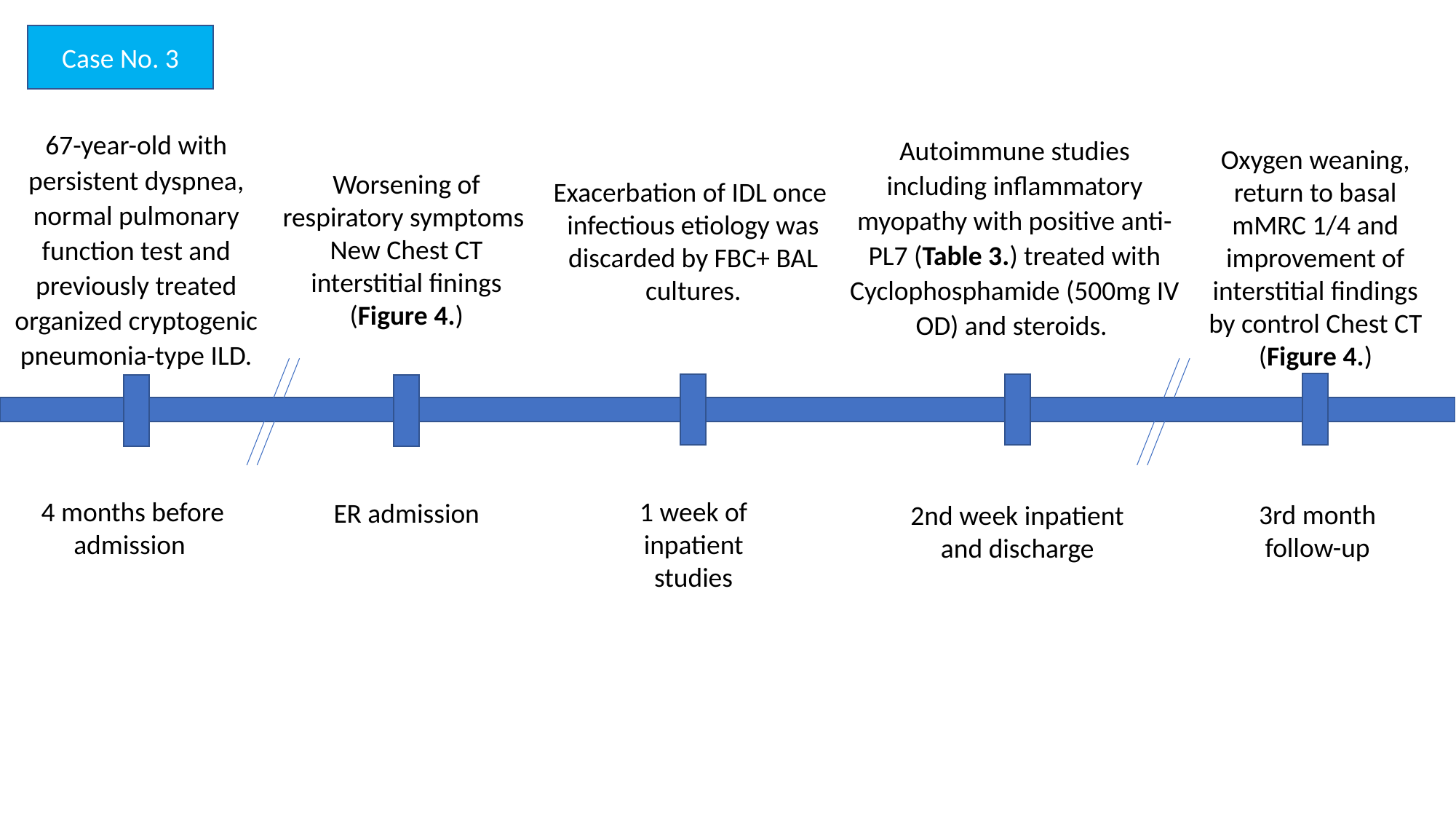

Case No. 3
67-year-old with persistent dyspnea, normal pulmonary function test and previously treated organized cryptogenic pneumonia-type ILD.
Autoimmune studies including inflammatory myopathy with positive anti-PL7 (Table 3.) treated with Cyclophosphamide (500mg IV OD) and steroids.
Oxygen weaning, return to basal mMRC 1/4 and improvement of interstitial findings by control Chest CT (Figure 4.)
Worsening of respiratory symptoms
New Chest CT interstitial finings (Figure 4.)
Exacerbation of IDL once infectious etiology was discarded by FBC+ BAL cultures.
1 week of inpatient studies
4 months before admission
ER admission
3rd month follow-up
2nd week inpatient
and discharge
